# Optical modeling-assisted characterization of dye-sensitized solar cells using TiO_2_ nanotube arrays as photoanodes

**DOI:** 10.3762/bjnano.5.102

**Published:** 2014-06-24

**Authors:** Jung-Ho Yun, Il Ku Kim, Yun Hau Ng, Lianzhou Wang, Rose Amal

**Affiliations:** 1 Nanomaterials Centre, School of Chemical Engineering, University of Queensland, St Lucia QLD 4072, Australia; 2 School of Mathematics and Physics, University of Queensland, St Lucia QLD 4072, Australia,; 3 School of Chemical Engineering, University of New South Wales, Kensington NSW 2052, Australia

**Keywords:** charge generation, dye-sensitized solar cells, generalized transfer matrix method, optical process, photocatalysis, TiO_2_ nanotubes

## Abstract

Photovoltaic characteristics of dye-sensitized solar cells (DSSCs) using TiO_2_ nanotube (TNT) arrays as photoanodes were investigated. The TNT arrays were 3.3, 11.5, and 20.6 μm long with the pore diameters of 50, 78.6, and 98.7 nm, respectively. The longest TNT array of 20.6 μm in length showed enhanced photovoltaic performances of 3.87% with significantly increased photocurrent density of 8.26 mA·cm^−2^. This improvement is attributed to the increased amount of the adsorbed dyes and the improved electron transport property with an increase in TNT length. The initial charge generation rate was improved from 4 × 10^21^ s^−1^·cm^−3^ to 7 × 10^21^ s^−1^·cm^−3^ in DSSCs based on optical modelling analysis. The modelling analysis of optical processes inside TNT-based DSSCs using generalized transfer matrix method (GTMM) revealed that the amount of dye and TNT lengths were critical factors influencing the performance of DSSCs, which is consistent with the experimental results.

## Introduction

Owing to its chemical durability, non-toxicity, and abundance, TiO_2_ has attracted great attention as a good photoelectrode material in dye-sensitized solar cells (DSSCs) [1–2]. In particular, the light harvesting capacity and dye loading, which are the important parameters affecting the amount of photogenerated electron charges for DSSCs performance, can be controlled by the structure and morphology of TiO_2_. For instance, roughness-increased surface-structured TiO_2_ photoelectrode layers, composed of sub-micrometer sized particulate, 2D-structured, or 1D-structured TiO_2_, can improve the light harvesting efficiency by promoting light scattering [3–5]. In addition, the electron transport or recombination rate could be influenced by physical properties such as porosity, morphology, crystallinity, and uniformity of the TiO_2_ structure [6–9]. TiO_2_ photoelectrode candidate materials with different structures and morphologies such as mesoporous TiO_2_, nanorods, nanotubes, nanosheets and hollow spheres have, therefore, been investigated to improve the performance of DSSCs by using various synthetic and modification methods [4–6,10–11].

Compared to the conventional DSSCs, which employ mesoporous TiO_2_ nanoparticles, vertically well-ordered TNT-based DSSCs presented an enhanced electron transport by efficiently reducing the recombination possibility of photogenerated charge carriers through minimizing the trapping sites that normally exist in the grain boundaries of randomly oriented TiO_2_ particulate films [12]. Consequently, this enhanced charge transport led to an improvement in the efficiency of light energy conversion. According to Zhu et al., as considering the charge collection efficiency between TiO_2_ nanoparticle-based and TNT-based DSSCs with comparable TiO_2_ thickness, the TNT-based DSSCs showed a 25 % higher charge collection efficiency than the TiO_2_ nanoparticle-based DSSCs. An outstanding optical effect induced by the well-ordered 1D-structure of the TNT array contributed to the improvement of the photovoltaic performance as well [5]. The light that penetrates into the open channels of the TNT array is scattered into deeper sites of the nanotubes, generating larger volume of excited electrons, thus enhancing the light harvesting efficiency in DSSCs [5,13]. Likewise, one of the strategies to improve the photovoltaic performance of the DSSCs is to increase the light harvest by tuning the optical processes in the devices. The optical processes in the solar cells include electric field intensity, charge generation rate, absorption and reflectance at all the interfaces formed between structural layers and electrodes in the devices [14–15]. These optical processes inside the devices can be modeled trough physical and optical parameters of the layers. In this work, the optical modeling study will provide critical insight for the experimental design by understanding the internal optical processes and parameters within the structure. Despite the promising optical properties of TNT arrays, the optical modeling of TNT-based DSSCs has rarely been studied. Thus the experimental work of TNT-based DSSCs coupled with optical modeling will be a good platform to understand the photovoltaic performance of the solar cells. The optical modeling result will provide the important information in assigning major contributing factors in the improvement of DSSC performance.

In this work, we present a comprehensive study on the ruthenium-based N719 dye-sensitized solar cells using TNT photoanodes through experimental work coupled with optical modeling analysis. The photovoltaic performances and electron transport properties of the fabricated DSSCs with different thickness of the TNT photoanodes are investigated. The simplified standard structures under the experimental condition are simulated by using a generalized transfer matrix method (GTMM) [15–16]. The comparison of the experimental results with the optical modeling results presents how charge generation and charge transport are associated with the unique morphological property of 1D-TNT photoanodes when enhancing the photovoltaic performance.

## Results and Discussion

### TNT-based N719 dye-sensitized solar cells

**Figure 1:**
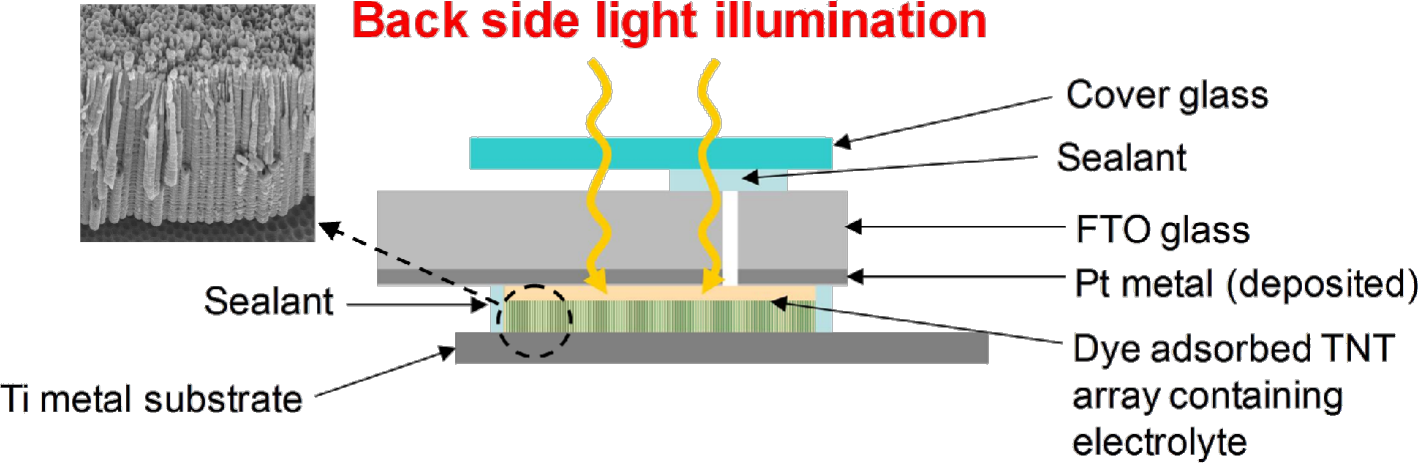
Schematic diagram of TNT-based DSSCs under back side light illumination (Inset SEM image indicates well-ordered TNT arrays prepared by anodization).

Prior to fabricating DSSCs, the anodization condition for TNT arrays as photoelectrodes was determined. The lengths of TNT arrays employed in the DSSCs fabrication were 3.3, 11.5, and 20.6 μm with different pore diameters of 50, 78.6, and 98.7 nm, respectively. In Figure 1, TNT-based DSSCs are operated by harvesting light illuminated from a back side passing through Pt-deposited FTO glass, and subsequently the penetrated light is absorbed by dye-sensitized well-ordered TNT arrays.

**Figure 2:**
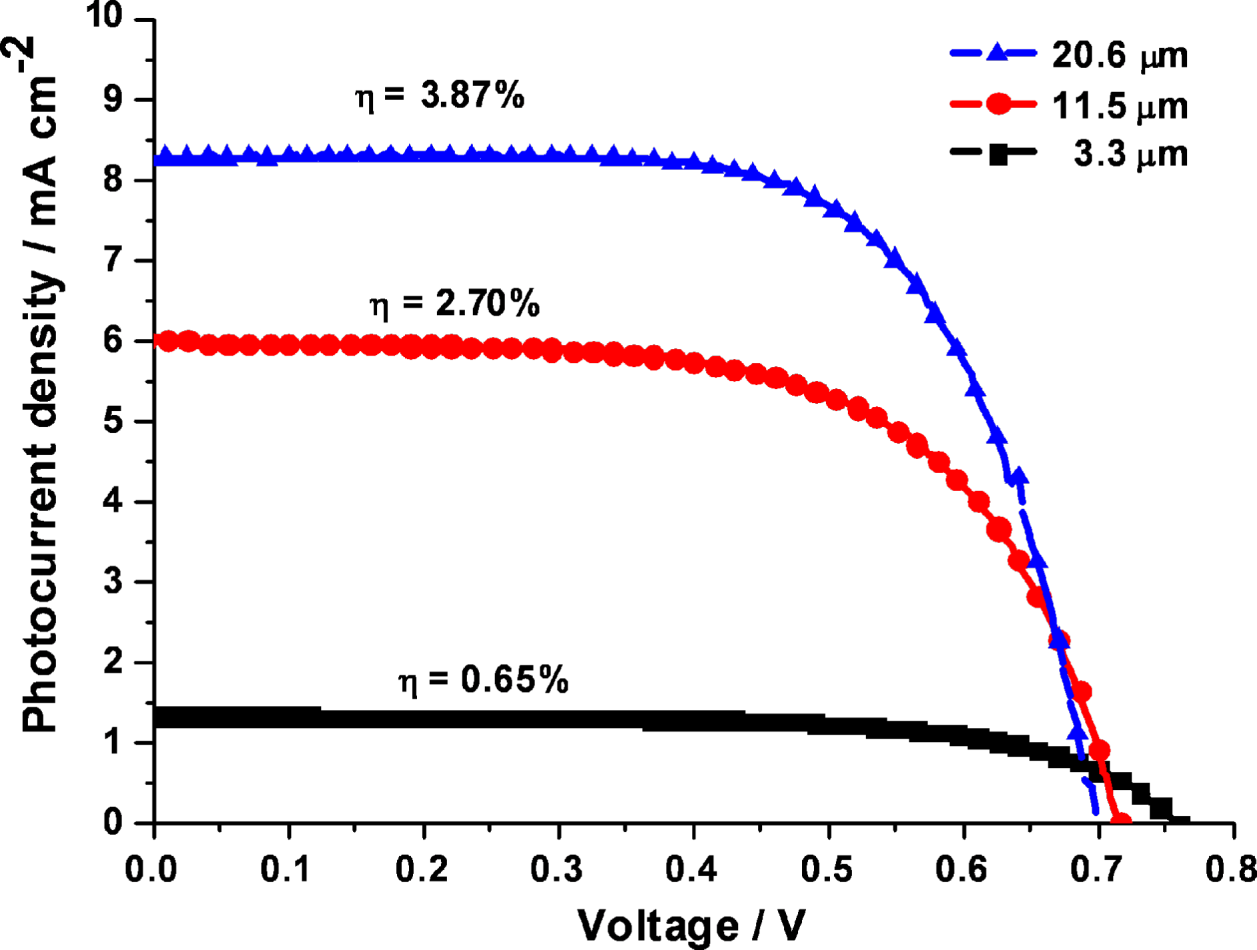
Photocurrent–voltage characteristics of N719-DSSCs fabricated by using 3.3 μm, 11.5 μm, and 20.6 μm TNT arrays under an AM 1.5 solar simulator (100 mW·cm^−2^).

In Figure 2, the photocurrent density–voltage curves for the TNT-based N719 DSSCs are shown depending on 3.3, 11.5, and 20.6 μm long TNT arrays as photoelectrodes. The DSSC with 3.3 μm long TNT arrays shows a short-circuit density (*J*_sc_) of 1.32 mA·cm^−2^, an open-circuit voltage (*V*_oc_) of 0.76 V, and a fill factor (FF) of 0.65, with a solar energy conversion efficiency of 0.65%. Meanwhile, 11.5 and 20.6 μm long TNT array-based DSSCs exhibited increases in *J*_sc_ to 6.02 mA·cm^−2^ (FF = 0.62) and 8.26 mA·cm^−2^ (FF = 0.67), respectively, whereas their *V*_oc_ values were reduced to 0.72 and 0.70 V, respectively. Consequently, in comparison with the photovoltaic performances of the 3.3 μm long TNT-based DSSC, these photovoltaic performances led to enhanced solar energy conversion efficiencies of 2.7% for the 11.5 μm long TNT array-based DSSC and 3.87% for the 20.6 μm long TNT array-based DSSC. Considering the results in Figure 2 and Table 1, the improved *J*_sc_ with the increase in the lengths of TNT arrays can be attributed to a larger surface area available for the adsorption of larger amounts of dye (Table 1), thus leading to the large amount of photogenerated electrons. Meanwhile, the decreased *V*_oc_ with the increase in the tube lengths can be explained by the electron dilution effect. The intensity of the penetrated light gradually decreases with an increase in the thickness of an electrode. Therefore, as the tube length increases, the excessive electron density becomes lower, resulting in a lower *V*_oc_. The higher series resistance of a longer tube length also influences the reduction of photovoltage [17–19].

**Table 1:** Photovoltaic performances of N719-DSSCs using different TNT arrays under an AM 1.5 solar simulator.

TNT length (μm)	*V*_oc_ (V)	*J*_sc_ (mA·cm^−2^)	FF	efficiency η (%)	dye loading (μM·cm^−2^)	pore volume (× 10^−5^ cm^3^)

3.3	0.76	1.32	0.65	0.65	20	5.94
11.5	0.72	6.02	0.62	2.70	38	12.36
20.6	0.70	8.26	0.67	3.87	59	28.24

**Figure 3:**
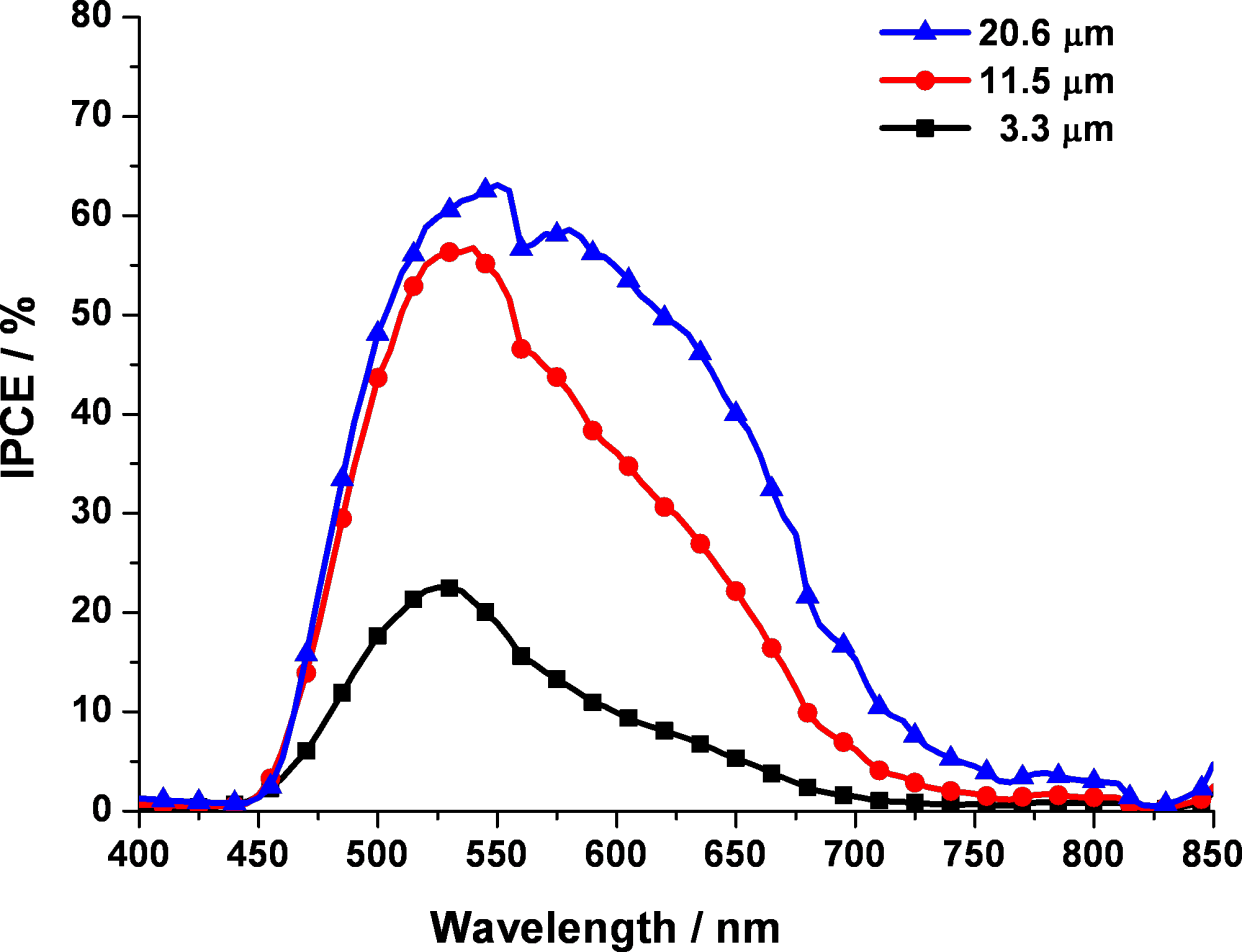
IPCE spectrum of the N719-DSSCs fabricated by using 3.3 μm, 11.5 μm, and 20.6 μm TNT arrays.

As seen in Figure 3, under the short-circuit condition, the incident photon-to-current conversion efficiency (IPCE) measurement was performed on the samples characterized in Figure 2. As the primary IPCE peak of N719-dye sensitized devices is usually observed at a wavelength of approximately 530–550 nm, the IPCE value of the 3.3 μm long TNT array was around 23% in this wavelength range. The increase in tube lengths to 11.5 and 20.6 μm led to the enhancement of the IPCE values to 57% and 63%, respectively. Below 500 nm the IPCE values were rapidly decreasing due to the reduced light absorption by the reflection effect of the electrolyte layer and the Pt deposited FTO glass in the counter electrode through back side illumination [20]. The steady increase in IPCE with longer tube lengths is attributed to the increase in *J*_sc_ by the increased amount of dyes adsorbed on the longer TNT arrays, followed by a further increase in the overall energy conversion efficiency. In principle, the IPCE depends on the light harvesting efficiency (LHE), the efficiency of electron injection from the photoexcited dye into the TiO_2_ conduction band (η_inj_), and the efficiency of charge collection at contacts (η_cc_) (Equation 1).

[1]
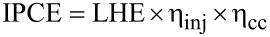


Herein, the LHE is determined by the amount of adsorbed dye, which is proportional to the tube length, and the light scattering effect that depends on film thickness and morphology [5,21–22]. Zhu et al. studied the differences of LHE between TNT-based DSSCs and TiO_2_ nanoparticle-based DSSCs. With comparable dye coverage and redox electrolyte composition on the different TiO_2_ electrodes, the TNT-based DSSCs showed an enhanced LHE value, which was ascribed to enhanced channeled light scattering in the TNT array with respect to that in the TiO_2_ nanoparticulate film [5]. Taking these results into account, the TNT morphology is beneficial to enhance the photovoltaic performances in the DSSCs by facilitating light scattering effects while enabling the manipulation of the tube length to accommodate for larger amounts of dye.

**Figure 4:**
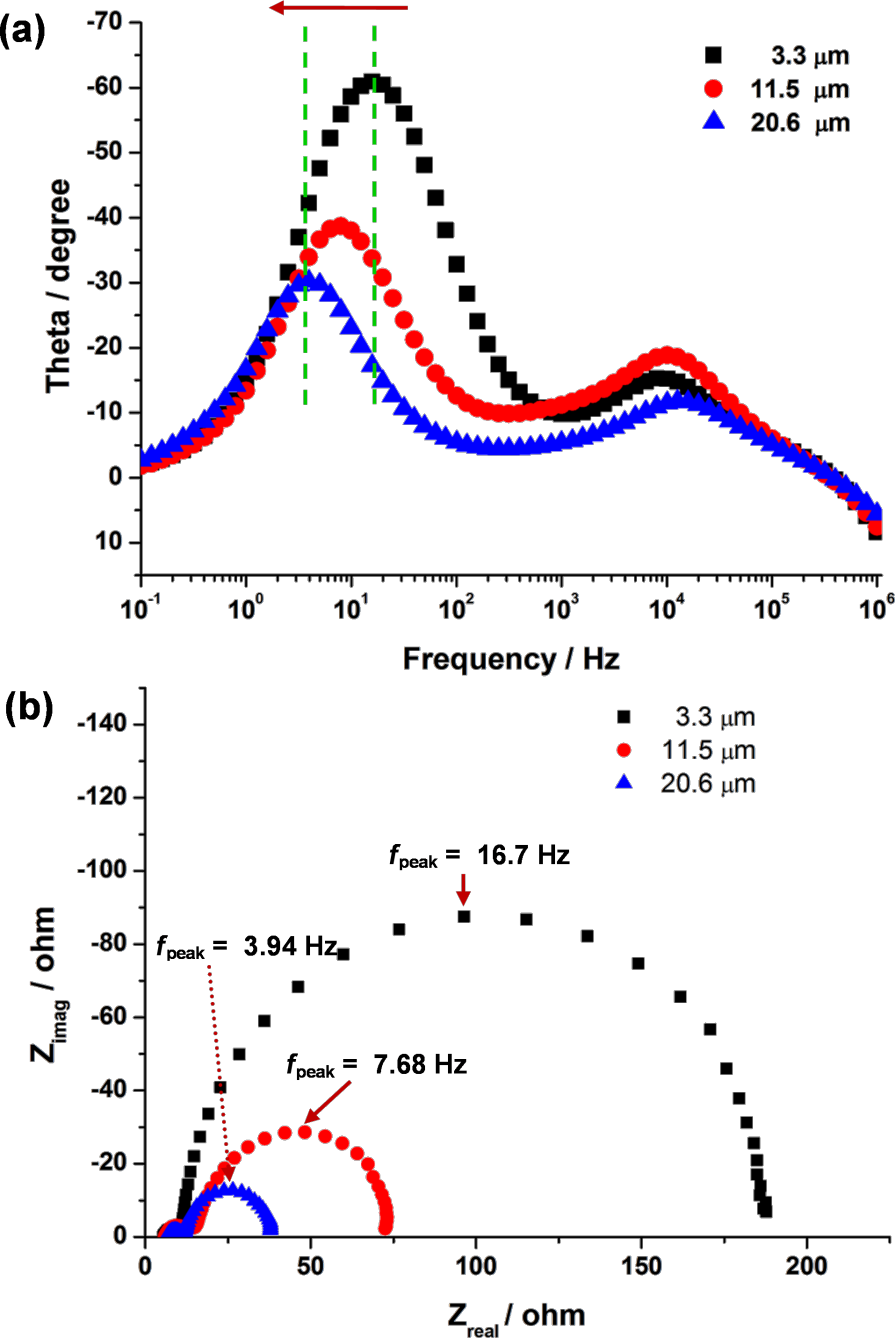
Experimental impedances of the N719-DSSCs fabricated using 3.3 μm, 11.5 μm, and 20.6 μm TNT arrays. (a) Bode phase plots and (b) Nyquist plots.

For the understanding and characterization of the fundamental photovoltaic parameters of the DSSCs, electrochemical impedance spectroscopy (EIS) offers valuable information. Figure 4 shows the Bode phase plots and the Nyquist plots obtained from electron transfer at the TiO_2_ and electrolyte interface under a solar simulator of AM 1.5. Figure 4a shows the negative shift of the frequencies of the main peaks with an increase in the lengths of TNT arrays. The peak frequencies were 16.7, 7.68, and 3.94 Hz with 3.3, 11.5, and 20.6 μm long TNT-based DSSCs, respectively. These peak frequencies in the Bode plot and the large semicircles in the Nyquist plot seen in Figure 4b are derived from the charge transfer reaction at the dye-sensitized TNT and electrolyte interface, whereas the smaller semicircle of the two semicircles in the Nyquist plots is attributed to the redox reaction at the electrolyte and counter electrode interface. In the meantime, the series resistance values of the DSSCs using 3.3, 11.5, and 20.6 μm long TNT arrays were 5.48, 5.86, and 6.51 Ω, respectively, indicating the series resistance obtained from EIS analysis is closely related to the thickness of the TiO_2_ films. It is clear that the series resistance increases with the increase in the length of the TNT arrays. This is confirmed by the result that the low *V*_oc_ value observed for the DSSC, which uses the long TNT array, shown in Figure 2, was due to the high series resistance. From the EIS results, furthermore, the electron lifetime of the 20.6 μm long TNT-based DSSCs with a lower peak frequency was longer than those of DSSCs using shorter TNT arrays (3.3 and 11.5 μm in length) with relatively high peak frequencies (Equation 2). The elongation of electron lifetime in the longer TNT arrays can be attributed to an increase in the electron retention time, accompanied by an increase in electron diffusion length from Equation 3.

[2]
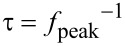


[3]
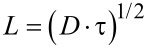


where τ, *f*_peak_, *L*, and *D* represent the electron lifetime in TiO_2_, the peak frequency of a large semicircle in Figure 4a, the electron diffusion length, and the diffusion coefficient, respectively.

### Optical modeling of TNT-based dye-sensitized solar cells

The generalized transfer matrix method (GTMM) has been applied to calculate and analyze the interference effect by multi-layers in solar cells [15]. The optical modeling using GTMM provides reliable information about the spatial distribution of the electric field intensity and the internal light absorption efficiency of the solar cells with mixed coherent and incoherent multi-layers [16]. The electric field intensity and charge generation rate as a function of the thickness of multi-layers and light fraction intensity were calculated based on Equations 4–6 according to Burkhard et al. [15]. For this, the only needed term of the optical constants is the imaginary part, *k*.

[4]
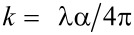


where λ is the wavelength of light and α the absorption coefficient. α is related to the optical density and the transmitted intensity by,

[5]
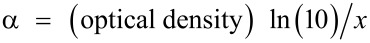


[6]
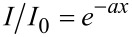


where *I/I*_0_ is the fraction of light that remains after passing through the film and *x* is the layer thickness.

**Figure 5:**
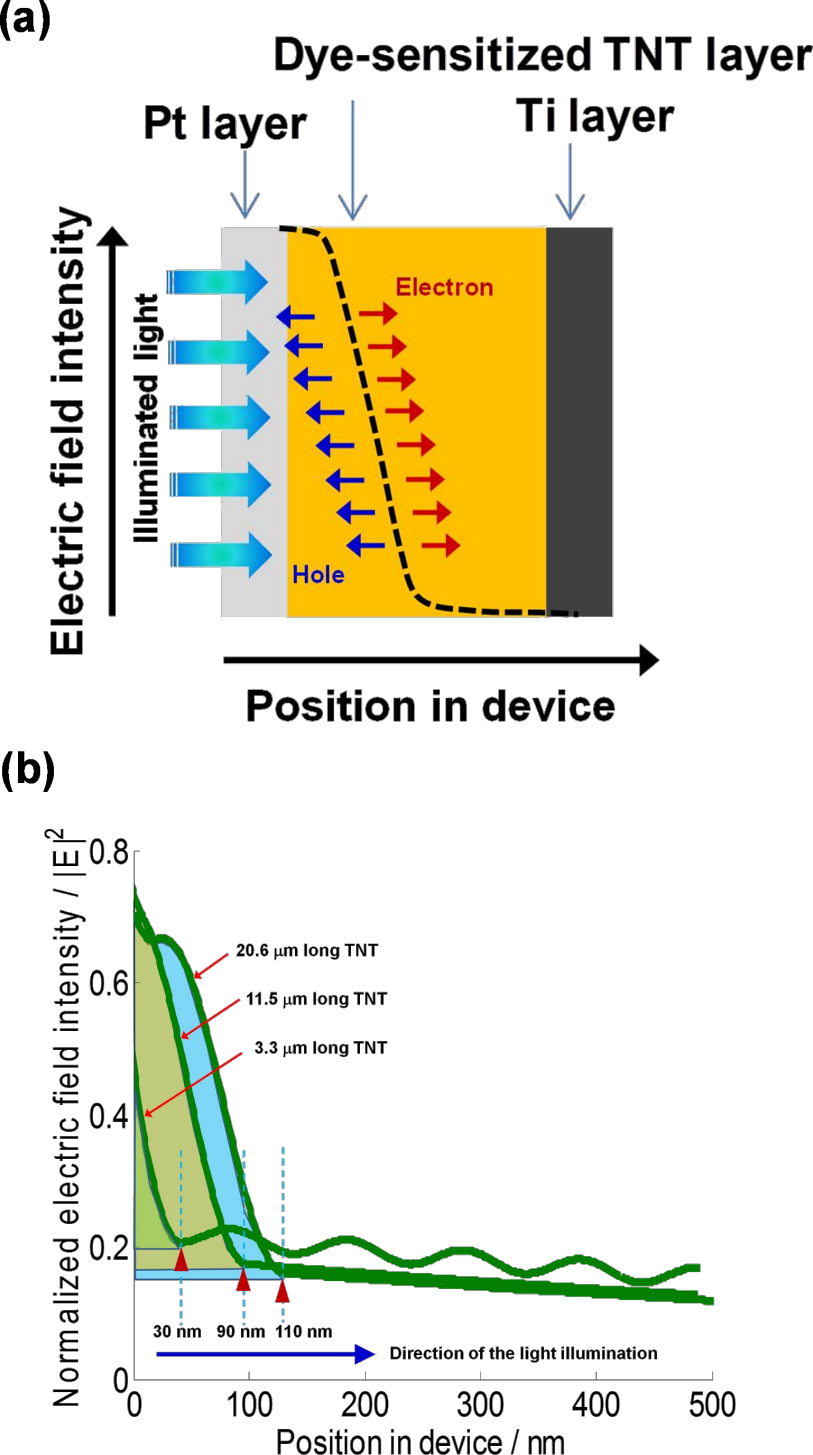
(a) A schematic diagram of charge separation driven by the electric field intensity as a function of layer thickness and (b) Electric field intensity of DSSCs with different TNT lengths calculated by using GTMM. (The onset values, 30 nm, 90 nm, and 110 nm, are electric field-valid layer thickness. That is the distance from the Pt layer.)

Figure 5 shows the calculated electric field intensity of the DSSCs with different TNT lengths by using GTMM. The electric field formed between active layers of solar cells triggers the charge separation of electron and hole generated in the solar cell system. The electric field intensity in Figure 5a shows the behavior of charge separation occurring at an interface between multi-layers as a function of the position in the device. The position in the device refers to the distance from the first layer (Pt layer) illuminated by light. Herein, for the modeling analysis, the configuration of DSSCs was simplified to three main layers: Pt layer, dye-sensitized TNT layer, and Ti foil, which are in the order of light contact under back-side light illumination. Figure 5b shows the electric field intensity of DSSCs when 3.3 μm, 11.5 μm, and 20.6 μm long TNT arrays are applied as a photoanode. The illuminating light has a wavelength of 550 nm, which is a primary light absorption peak of N719 dye, corresponding to the IPCE result in Figure 3. Under the same illumination of 550 nm wavelength, the magnitude of the electric field intensity increased with the increase in the lengths of the TNT. The magnitude of the dye/TNT active region in DSSCs using 20.6 μm long TNT arrays was the largest as seen in the blue-colored integrated area of Figure 5b, where the DSSCs with 20.6 μm long TNT arrays is expected to have the best light absorption efficiency at the wavelength of 550 nm. In addition, the valid layer thickness governed by the electric field intensity in DSSCs is a function of the TNT lengths: they were 30, 90 and 110 nm for 3.3, 11.5 and 20.5 μm long TNT arrays, respectively, as determined by the onset points of the position in device. Considering the fact that the employed TNT arrays are on the micrometer-scale, the valid layer thickness at the nanometer-scale indicates the active layer in the DSSCs is mainly controlled by the adsorbed dye on the TNT array. The valid layer thickness is likely to be the imaginary thickness of the adsorbed dye layer in terms of optical modeling although the adsorbed dye is usually considered as a volume. Therefore, with the longer TNT array, the larger magnitude of the electric field intensity and the thicker electric field-valid layer contribute to the higher light harvesting with an enhanced charge separation. This is well matched with the *J*_sc_ and IPCE results and this explanation is supported by the modeled results which will be discussed later.

**Figure 6:**
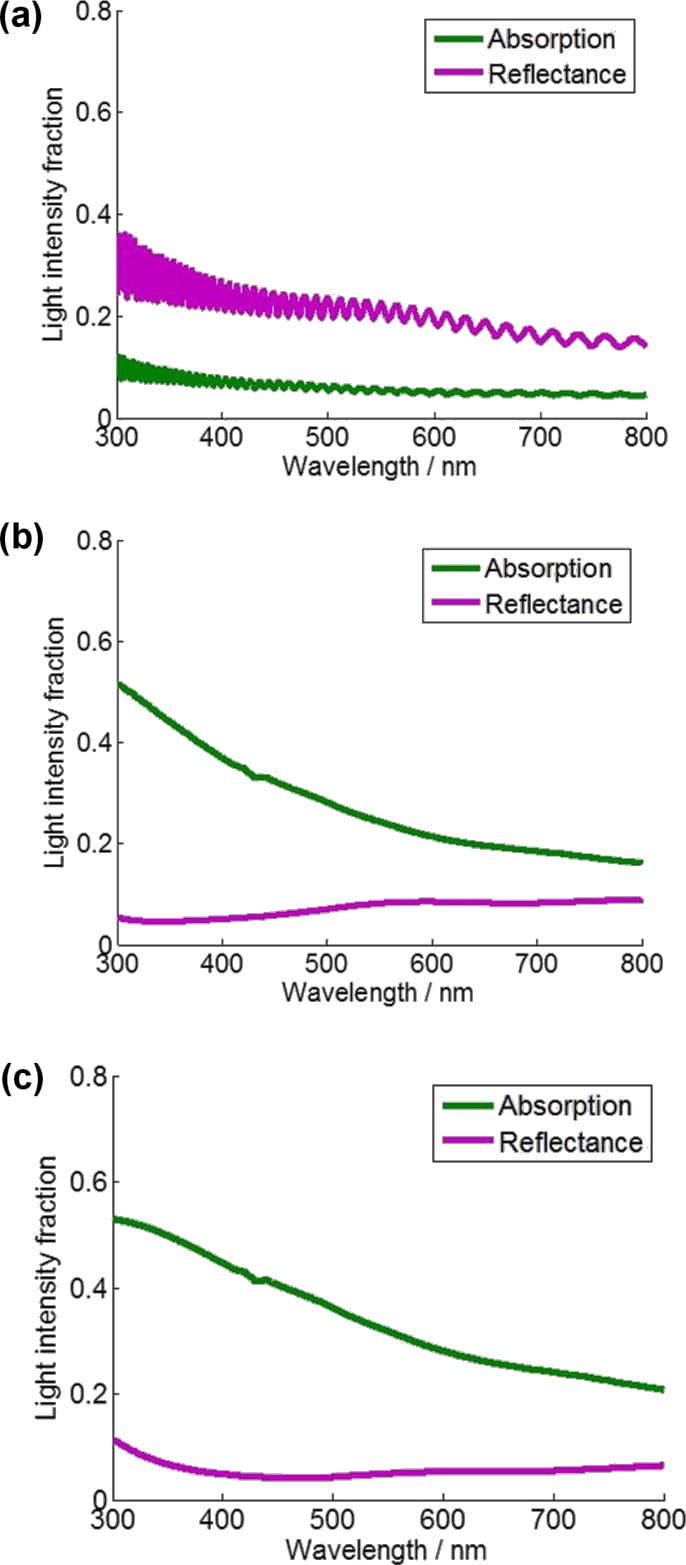
Calculated absorption and reflectance of the DSSCs with different TNT lengths by using GTMM: (a) 3.3 μm long TNT array, (b) 11.5 μm long TNT array, and (c) 20.6 μm long TNT array.

**Figure 7:**
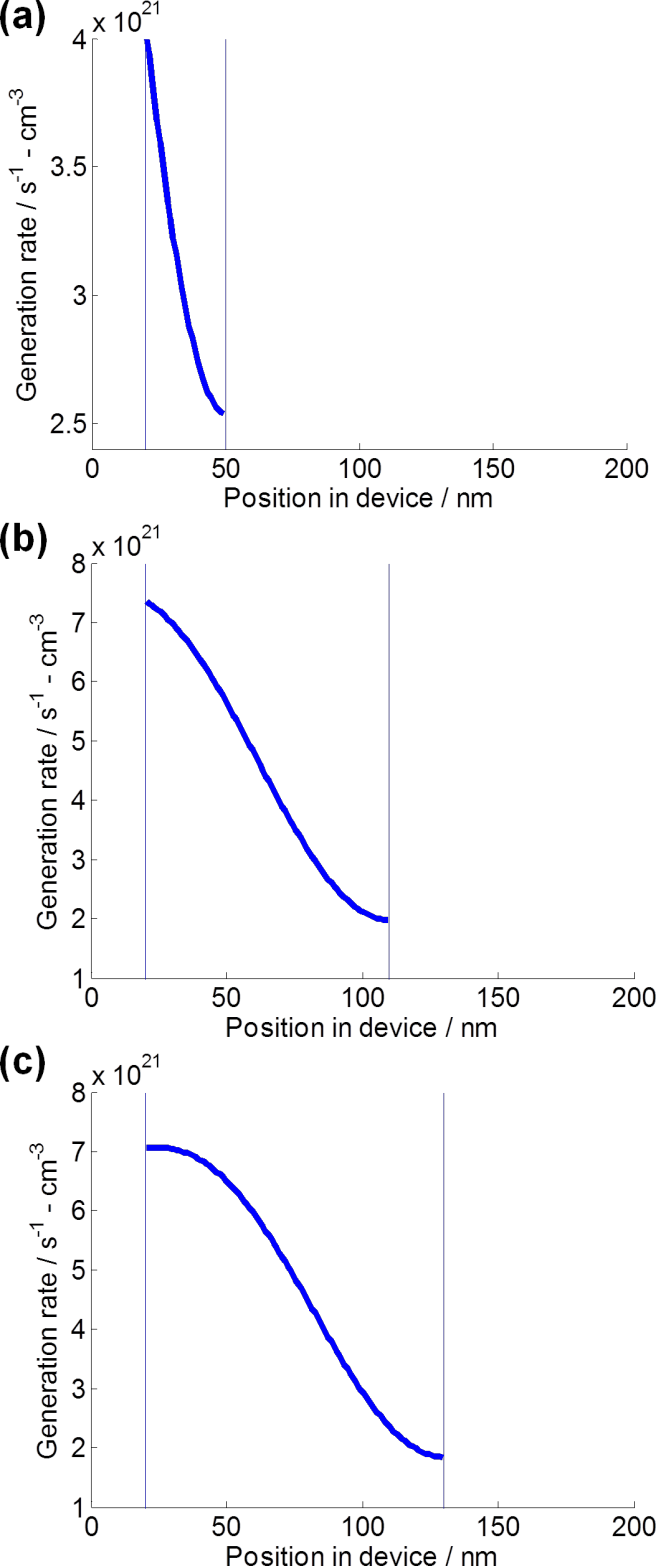
Calculated charge generation rate of the DSSCs with different TNT lengths using GTMM under 1 sun condition (100 mW·cm^−2^). Charge generation rate for DSSCs with (a) 30 nm thick electric field-valid layer of a 3.3 μm long TNT array, (b) 90 nm thick electric field-valid layer of a 11.5 μm long TNT array, and (c) 110 nm thick electric field-valid layer of a 20.6 μm long TNT array.

Figure 6 shows the light intensity fraction of absorption and reflectance of the TNT-based DSSCs by using GTMM with 3.3 μm, 11.5 μm, and 20.6 μm long TNT arrays. As indicated in Figure 6a, the rate of reflection of light is higher than the rate of absorption of the dye, which indicates that incident light is lost by reflection. This clearly supports that the IPCE value of DSSCs with the 3.3 μm long TNT array was lower at all incident wavelengths than those of longer TNT-employed DSSCs (Figure 3). Additionally, the fluctuating light fraction intensity result of 3.3 μm long TNT-DSSCs presents the active layer consisting of dye and TNT array did not absorb the penetrating light effectively, confirmed by the experimental result of the lowest *J*_sc_ (1.32 mA·cm^−2^). In contrast, with longer TNT lengths of 11.5 μm and 20.6 μm, the reflectance and absorbance shows significantly reversed changes (Figure 6b and Figure 6c). As the light is illuminated into DSSCs using 11.5 μm and 20.6 μm long TNT arrays, the reflectance dramatically decreases and the absorption significantly increases, while the magnitude of light fraction intensity of the 20.6 μm long TNT array is slightly higher than that of the 11.5 μm long TNT array. These light fraction intensity results reflect the charge generation rate with different TNT lengths in Figure 7.

While the major contribution of GTMM is to understand the electric field intensity distribution, it can also be applied for calculating the amount of generated charges in the photo-active layer [23]. In all cases, charge generation is mostly in the dye-sensitized active layer. In Figure 7, the charge generation rate is calculated in the range of the electric field-valid layer thicknesses obtained from Figure 5b. There is an initially high rate of charge generation which gradually decreases. This is due to an increase in recombination and a decrease in light absorption with an increase in thickness of layer. With an increase of the electric field-valid layer thickness from 30 nm to 110 nm (Figure 5b), the initial rate of charge generation of 4 × 10^21^ s^−1^·cm^−3^ is boosted up to 7 × 10^21^ s^−1^·cm^−3^. While the initial charge generation rates of DSSCs using 11.5 μm long TNT arrays and 20.6 μm long TNT arrays are comparable, their slopes in Figure 7b and Figure 7c show different patterns. In Figure 7c, the generated charge is retained up to 50 nm of position in the device before it drops. In contrast, Figure 7b shows that the generated charge rate rapidly decreases within the range of the electric field-valid layer of 90 nm. Therefore, compared to *J*_sc_ (6.02 mA·cm^−2^) of DSSCs using 11.5 μm long TNT arrays, the higher *J*_sc_ (8.26 mA·cm^−2^) of DSSCs using 20.6 μm long TNT arrays was attributed to relatively high charge generation rate [24]. By comparing the calculated charge generation rates with experimental data such as IPCE and EIS, the enhancement of photovoltaic performance with higher *J*_sc_ is achieved from the improvement of charge generation rate facilitated by light harvest, charge separation and electron lifetime.

## Conclusion

DSSCs using 1D-TNT photoanodes have been comprehensively studied by optical modeling-assisted characterization. The photovoltaic performance of DSSCs with longer TNT lengths was significantly enhanced through an increase in *J*_sc_. The amount of dye and IPCE analysis confirmed the increase in *J*_sc_ was due to the increased light harvest rate, supported by the charge generation rate calculated from optical modeling using GTMM. The increase in *J*_sc_ was also due to the excellent charge transport property of 1D-TNT structured photoanodes accompanying effective electron–hole charge separation and longer electron lifetime, which were confirmed by EIS analysis and the simulated electric field intensity. Therefore, our characterization approach employing optical modeling contributes to a deeper understanding of the improved light harvesting and charge transport properties observed in the solar cell devices using 1D-TNT photoanodes.

## Experimental

### Fabrication of TNT-based DSSCs

Under the anodization conditions of 60 V with ethylene glycol containing 0.5 wt % NaF and 5 wt % water, TNT arrays (6 × 6 mm^2^) with different lengths and pores were obtained by various anodizing durations of 1, 5, and 15 h [25]. The TNT arrays were immersed in 0.04 M of TiCl_4_ at 70 °C for 30 min followed by rinsing with water. The TiCl_4_ treated TNT was calcined at 450 °C for 3 h and reheated to 450 °C for 30 min if not immediately used. For N719-DSSCs, the TNT array was soaked in 0.3 mM N719 (*cis*-diisothiocyanato-bis(2,2′-bipyridyl-4,4′-dicarboxylato)ruthenium(II) bis(tetrabutylammonium)) dye solution in anhydrous acetonitrile for 18 h. N719 compound was purchased from Sigma-Aldrich. A sandwich-type DSSC was assembled using the dye-sensitized TNT array onto the Ti foil (20 × 20 mm^2^) as a photoelectrode and platinum-deposited fluorine-doped tin oxide (FTO) glass (20 × 15 mm^2^, Asahi, *R*_s_ ≤ 8 Ω·sq^−1^) as a counter electrode separated by a sealant (Surlyn 60 µm thickness, Solaronix). The electrolyte was a mixture of 0.1 M LiI, 0.6 M 1, 2-dimethyl-3-propylimidazolium iodide (DMPII), 0.03 M I_2_ and 0.5 M *t*-butylpyridine (*t*-BP) in acetonitrile. The electrolyte was injected to the cell through a hole (diameter 1 mm) drilled through the counter electrode with the aid of a vacuum. The fabricated active area in the single cell was 0.16 cm^2^ (4 × 4 mm^2^).

### Characterization of TNT-based DSSCs

The photovoltaic performances of the DSSCs were measured using a Keithley 2400 source measure unit under a calibrated AM 1.5 solar simulator (Oriel) at 100 mW·cm^−2^ light intensity. Incident photon-to-current conversion efficiency (IPCE) spectra of the devices were measured under a Xe-lamp (Newport 66902) equipped with a monochromator (Newport 74125). The light illumination was concentrated onto a spot smaller than the cell area. The short-circuit current response of the devices was measured in 5 nm steps using a Keithley 2400 source measure unit. The amount of adsorbed dye concentration was determined by measuring the absorbance of dye solution desorbed from the surface of the TNT array in basic solution. In order to desorb dyes, N719 dye-adsorbed TNT arrays were immersed in a 0.1 M NaOH in water for about 40 min. The absorbance measurement was performed using UV–vis spectrophotometer (Cary 300, Varian). The electrochemical impedance spectroscopy (EIS) measurements were performed by illuminating the DSSCs with a AM 1.5 solar simulator calibrated at 100 mW·cm^−2^ at open-circuit conditions between 0.1 Hz and 100 kHz with an AC amplitude of ± 10 mV using a Gamry Reference 600 instrument.

### Transfer matrix method analysis for TNT-based DSSCs

For transfer matrix method analysis for TNT-based DSSCs, the ellipsometry measurement system was used to get the optical constants such as refractive index (*n*) and extinction coefficient (*k*). Prior to the ellipsometry measurement, the individual layers inside the DSSCs were prepared on a cleaned Si wafer of 2.5 × 2.5 cm^2^. The Si wafer was sequentially cleaned in acetone, isopropyl alcohol and milli-Q water with 5 min sonication, respectively. The N719 dye and Pt layers were deposited on the Si wafer using spin coating under the condition of 2000 rpm for 60 s. For the TNT arrays, TNT arrays with three different lengths (3.3 μm, 11.5 μm, and 20.6 μm) were transplanted from Ti foil substrate to the Si wafer with 0.04 M TiCl_4_ solution and then calcined at 450 °C for 3 h. The Ti foil was cut to 2.5 × 2.5 cm^2^. Optical constants were obtained by using a VUV-VASE ellipsometer system (J. A. Woollam Co., Inc.). The transfer matrix program and procedure were adopted from the literature [15–16].
